# Supplementary material for: Multiomic ALS signatures highlight subclusters and sex differences suggesting the MAPK pathway as therapeutic target
Source: Nat Commun. 2024 Jun 7;15:4893. doi: 10.1038/s41467-024-49196-y (PMC11161513; doi:10.1038/s41467-024-49196-y)
Supplement: Supplementary file 4 — Supplementary Data 1-16 [file 41467_2024_49196_MOESM4_ESM.zip › Supplementary Data 1-16/Supplementary Data - legends.docx]

**Supplementary Data**

**Supplementary Data 1**

Comprehensive multiomic characterization of the human postmortem prefrontal cortex (Brodmann area 6), comparing 51 patients with neuropathologically confirmed amyotrophic lateral sclerosis (ALS) to 50 control patients. This table provides individual descriptions of the brain samples, aimed at elucidating early molecular changes in sporadic ALS (sALS).

**Supplementary Data 2**

Genetic analysis of human samples, including gene panel and C9orf72 repeat expansion analyses. This table details the genetic profiles assessed in the study's human brain samples to investigate genetic variations associated with amyotrophic lateral sclerosis (ALS).

**Supplementary Data 3**

Differential expression (DE) results from transcriptomic analysis. DE analysis was performed using DESeq2, employing a two-sided test and multiple test correction via the Benjamini-Hochberg method. This table summarizes the significantly differentially expressed genes identified in the study.

**Supplementary Data 4**

Downsampling bootstrapping analysis for transcriptomics data. This table presents the results of bootstrapping analyses conducted to assess the stability and reliability of transcriptomic findings under varied sample sizes.

**Supplementary Data 5**

Differential expression analysis of differentially alternatively spliced (DAS) events. This table lists the results highlighting significant changes in alternative splicing patterns across samples analyzed in the study. Differential testing was performed using SUPPA2 (two-sided empirical p-values) with Benjamini-Hochberg multiple test correction.

**Supplementary Data 6**

Pathway enrichment results for differentially alternatively spliced (DAS) events in human ALS patients and mouse models. This table displays the pathways enriched with DAS events, providing insights into the potential biological processes affected in both human and mouse models. Enrichment was performed using clusterProfiler enrichGO function with Benjamini-Hochberg multiple test correction.

**Supplementary Data 7**

Full pathway enrichment results for human transcriptomics data. This table provides comprehensive details of the enriched biological pathways identified in the differential expression analysis, offering insights into the molecular mechanisms underlying the studied condition. Gene-set enrichment was performed using clusterProfiler with multiple test correction via the Benjamini-Hochberg method.

**Supplementary Data 8**

Gene Ontology (GO) enrichments for all Weighted Gene Co-expression Network Analysis (WGCNA) modules. Right-tailed Fisher’s Exact test with Benjamini-Hochberg correction was used to compute statistics.

**Supplementary Data 9**

Differential expression analysis of miRNA using DESeq2 with a two-sided test. The analysis included multiple test corrections using the Benjamini-Hochberg method to control for false discovery rate.

**Supplementary Data 10**

Differential expression results for miRNA clusters. To assess the significance of miRNA expression changes within specific human disease clusters, we conducted a cluster-specific differential expression analysis using a two-sided Wald test within DESeq2 and applied multiple test corrections with the Benjamini-Hochberg method.

**Supplementary Data 11**

Differential expression analysis of proteomics data using the limma package, employing a two-sided test with multiple test corrections using the Benjamini-Hochberg method. This table details the significantly differentially expressed proteins identified, providing insights into the proteomic alterations associated with the condition studied.

**Supplementary Data 12**

REVIGO analysis of proteomics data: List of top Gene Ontology (GO) terms per cluster. This table presents the most significant GO terms for each proteomic cluster, highlighting key biological processes, cellular components, and molecular functions enriched in the dataset.

**Supplementary Data 13**

List of valid triplets (humans) and quadruplets (mouse). This table presents identified sets of three and four entities, respectively. For proteomics and phosphoproteomics, DE was performed using the Empirical Bayes method from Limma. DE analysis for transcriptomics and microRNA was performed using a 2-sided Wald test through DESeq2. The p values were corrected using Benjamini-Hochberg (BH) method.

**Supplementary Data 14**

MOFA (Multi-Omics Factor Analysis) Features. This table details the features identified through MOFA and highlights the key factors and their associated features across different omics data sets.

**Supplementary Data 15**

Mouse enrichment results with multiple test correction using the familywise-error rate  (FWER). This table displays the results of pathway and gene set enrichment analyses performed on mouse data. It includes the statistically significant pathways and sets after applying FWER to correct for multiple testing, offering insights into the biological processes most affected in the mouse model of the study

**Supplementary Data 16**

Parameters used for Weighted Gene Co-expression Network Analysis (WGCNA). This table lists all the parameters and settings used in the WGCNA process, including (minimum number of genes in a module, height to merge modules at, correlation threshold for module selection and soft power threshold).
