# Supplementary material for: Multiomic ALS signatures highlight subclusters and sex differences suggesting the MAPK pathway as therapeutic target
Source: Nat Commun. 2024 Jun 7;15:4893. doi: 10.1038/s41467-024-49196-y (PMC11161513; doi:10.1038/s41467-024-49196-y)
Supplement: Supplementary file 5 — Reporting Summary [file 41467_2024_49196_MOESM5_ESM.pdf]

nature portfolio

Corresponding author(s): Paul Lingor

Last updated by author(s): 16.05.2024

Reporting Summary

Nature Portfolio wishes to improve the reproducibility of the work that we publish. This form provides structure for consistency and transparency in reporting. For further information on Nature Portfolio policies, see our Editorial Policies and the Editorial Policy Checklist.

Statistics

For all statistical analyses, confirm that the following items are present in the figure legend, table legend, main text, or Methods section.

n/a	Confirmed	
☐	☒	The exact sample size (*n*) for each experimental group/condition, given as a discrete number and unit of measurement
☐	☒	A statement on whether measurements were taken from distinct samples or whether the same sample was measured repeatedly
☐	☒	The statistical test(s) used AND whether they are one- or two-sided<br>*Only common tests should be described solely by name; describe more complex techniques in the Methods section.*
☐	☒	A description of all covariates tested
☐	☒	A description of any assumptions or corrections, such as tests of normality and adjustment for multiple comparisons
☒	☐	A full description of the statistical parameters including central tendency (e.g. means) or other basic estimates (e.g. regression coefficient) AND variation (e.g. standard deviation) or associated estimates of uncertainty (e.g. confidence intervals)
☐	☒	For null hypothesis testing, the test statistic (e.g. *F*, *t*, *r*) with confidence intervals, effect sizes, degrees of freedom and *P* value noted<br>*Give P values as exact values whenever suitable.*
☐	☒	For Bayesian analysis, information on the choice of priors and Markov chain Monte Carlo settings
☐	☒	For hierarchical and complex designs, identification of the appropriate level for tests and full reporting of outcomes
☐	☒	Estimates of effect sizes (e.g. Cohen's *d*, Pearson's *r*), indicating how they were calculated

Our web collection on statistics for biologists contains articles on many of the points above.

Software and code

Policy information about availability of computer code

Data collection	we did not develop new algorithms
Data analysis	data analysis was performed with publicly available tools and referenced accordingly, https://github.com/imsb-uke/MAXOMOD_Pipeline

For manuscripts utilizing custom algorithms or software that are central to the research but not yet described in published literature, software must be made available to editors and reviewers. We strongly encourage code deposition in a community repository (e.g. GitHub). See the Nature Portfolio guidelines for submitting code & software for further information.

Data

Policy information about availability of data

All manuscripts must include a data availability statement. This statement should provide the following information, where applicable:

- Accession codes, unique identifiers, or web links for publicly available datasets
- A description of any restrictions on data availability
- For clinical datasets or third party data, please ensure that the statement adheres to our policy

A data availability statement is included in the declarations section of the manuscript.

Research involving human participants, their data, or biological material

Policy information about studies with human participants or human data. See also policy information about sex, gender (identity/presentation), and sexual orientation and race, ethnicity and racism.

Reporting on sex and gender	included in the manuscript. Cohort numbers: ALS: N=16 females/35 males; Controls: N=28 females/22 males
Reporting on race, ethnicity, or other socially relevant groupings	not considered for this manuscript.
Population characteristics	Included in the manuscript. Samples from patients diagnosed with ALS compose the ALS cohort. Samples from persons without any signs of neurodegenerative diseases compose the control cohort.
Recruitment	Postmortem brain sample recruitment was done from four European Brain Banks: The Netherlands Brain Bank (NBB), London Neurodegenerative Diseases Brain Bank (London NDBB), Imperial College London - Multiple Sclerosis and Parkinson's Tissue Bank (ICL MS&PD TB), and the Oxford Brain Bank (OBB).
Ethics oversight	Ethical approval for the use of human tissues was obtained from the Ethics Committees of the University Medical Center Göttingen (2/8/18 AN) and the Technical University Munich (145/19 S-SR).

Note that full information on the approval of the study protocol must also be provided in the manuscript.

Field-specific reporting

Please select the one below that is the best fit for your research. If you are not sure, read the appropriate sections before making your selection.

[x] Life sciences [ ] Behavioural & social sciences [ ] Ecological, evolutionary & environmental sciences

For a reference copy of the document with all sections, see nature.com/documents/nr-reporting-summary-flat.pdf

Life sciences study design

All studies must disclose on these points even when the disclosure is negative.

Sample size	human N=101 (ALS: N=16 females/35 males; Controls: N=28 females/22 males). For each model, a total of 20 transgenic and non-transgenic mice were selected and balanced for condition and sex (TDP-43 model: 5 females vs. 5 males for transgenic and control cohorts; SOD1 model: 5 females vs. 6 males for the control cohort and 5 females vs. 4 males for the transgenic cohort; C9orf72 model: 6 females vs. 4 males for the control cohort and 4 females vs. 6 males for the transgenic cohort; FUS model: 5 females vs. 5 males for transgenic and control cohorts). Validation considered at least 3 mice per group (as detailed below)..
Data exclusions	Mouse: FUS (only RNA-seq): S78; FUS (only srna): S858
Replication	disclosed for the validation section, replication numbers declared in text for each experiment. For cell cultures experiments (Fig.5): neurite outgrowth and cell survival experiments: (n = 5 independent neuronal cultures); for western blot validations of pErk1/2/Erk1/2 experiments: (n = 3 different cultures); for validation in animal SOD1 transgenic animals - pMEK2 validation experiments (Fig. 6a): (Non-transgenic, n=6; SOD1, n=4 per group); Fig.6b-f - study in female and male SOD1 mice un/treated with trametinib (9 to 16 weeks): Dot blot analysis of pERK1/2 (female, n = 4 per group; male, n = 4 vehicle-treated mice; n = 5 trametinib-treated mice) (b), p62 (female, n = 3 vehicle-treated mice; n = 5 trametinib-treated mice; male, n = 5 per group) (c), insoluble SOD1 (female, n = 4 per group; male, n = 5 vehicle-treated mice; n = 4 trametinib-treated mice) (d), and ubiquitin (female, n = 4 per group; male, n = 5 per group) (e) in the spinal cord of SOD1 female and male mice treated with trametinib or vehicle; (f) SOD1 mice treated or not with trametinib. Data are mean ± SEM (female, n = 3 vehicle-treated mice and n = 5 trametinib-treated mice; male, n = 4 vehicle-treated mice and n = 5 trametinib-treated mice). Preclinical study with trametinib treatment in SOD1 female mice (fig. 6i-j): vehicle-treated mice n=8; trametinib-treated mice n=10). Plasma NfL levels (Fig.6k) - analyzed in SOD1 female mice, with and without trametinib treatment, at 16 and 19 weeks. Data are mean ± SEM (16 weeks: vehicle n=3, trametinib n=5; 19 weeks: vehicle n=5, trametinib n=6).
Randomization	no randomizations employed for cell culture experiments, measurements in mice were performed in a blinded and randomized fashion.,
Blinding	no blinding employed for cell culture experiments,, measurements in mice were performed in a blinded and randomized fashion.

Behavioural & social sciences study design

All studies must disclose on these points even when the disclosure is negative.

Study description	*Briefly describe the study type including whether data are quantitative, qualitative, or mixed-methods (e.g. qualitative cross-sectional, quantitative experimental, mixed-methods case study).*

Research sample	*State the research sample (e.g. Harvard university undergraduates, villagers in rural India) and provide relevant demographic information (e.g. age, sex) and indicate whether the sample is representative. Provide a rationale for the study sample chosen. For studies involving existing datasets, please describe the dataset and source.*
Sampling strategy	*Describe the sampling procedure (e.g. random, snowball, stratified, convenience). Describe the statistical methods that were used to predetermine sample size OR if no sample-size calculation was performed, describe how sample sizes were chosen and provide a rationale for why these sample sizes are sufficient. For qualitative data, please indicate whether data saturation was considered, and what criteria were used to decide that no further sampling was needed.*
Data collection	*Provide details about the data collection procedure, including the instruments or devices used to record the data (e.g. pen and paper, computer, eye tracker, video or audio equipment) whether anyone was present besides the participant(s) and the researcher, and whether the researcher was blind to experimental condition and/or the study hypothesis during data collection.*
Timing	*Indicate the start and stop dates of data collection. If there is a gap between collection periods, state the dates for each sample cohort.*
Data exclusions	*If no data were excluded from the analyses, state so OR if data were excluded, provide the exact number of exclusions and the rationale behind them, indicating whether exclusion criteria were pre-established.*
Non-participation	*State how many participants dropped out/declined participation and the reason(s) given OR provide response rate OR state that no participants dropped out/declined participation.*
Randomization	*If participants were not allocated into experimental groups, state so OR describe how participants were allocated to groups, and if allocation was not random, describe how covariates were controlled.*

Ecological, evolutionary & environmental sciences study design

All studies must disclose on these points even when the disclosure is negative.

Study description	*Briefly describe the study. For quantitative data include treatment factors and interactions, design structure (e.g. factorial, nested, hierarchical), nature and number of experimental units and replicates.*
Research sample	*Describe the research sample (e.g. a group of tagged Passer domesticus, all Stenocereus thurberi within Organ Pipe Cactus National Monument), and provide a rationale for the sample choice. When relevant, describe the organism taxa, source, sex, age range and any manipulations. State what population the sample is meant to represent when applicable. For studies involving existing datasets, describe the data and its source.*
Sampling strategy	*Note the sampling procedure. Describe the statistical methods that were used to predetermine sample size OR if no sample-size calculation was performed, describe how sample sizes were chosen and provide a rationale for why these sample sizes are sufficient.*
Data collection	*Describe the data collection procedure, including who recorded the data and how.*
Timing and spatial scale	*Indicate the start and stop dates of data collection, noting the frequency and periodicity of sampling and providing a rationale for these choices. If there is a gap between collection periods, state the dates for each sample cohort. Specify the spatial scale from which the data are taken*
Data exclusions	*If no data were excluded from the analyses, state so OR if data were excluded, describe the exclusions and the rationale behind them, indicating whether exclusion criteria were pre-established.*
Reproducibility	*Describe the measures taken to verify the reproducibility of experimental findings. For each experiment, note whether any attempts to repeat the experiment failed OR state that all attempts to repeat the experiment were successful.*
Randomization	*Describe how samples/organisms/participants were allocated into groups. If allocation was not random, describe how covariates were controlled. If this is not relevant to your study, explain why.*
Blinding	*Describe the extent of blinding used during data acquisition and analysis. If blinding was not possible, describe why OR explain why blinding was not relevant to your study.*

Did the study involve field work? ☐ Yes ☐ No

Field work, collection and transport

Field conditions	*Describe the study conditions for field work, providing relevant parameters (e.g. temperature, rainfall).*
Location	*State the location of the sampling or experiment, providing relevant parameters (e.g. latitude and longitude, elevation, water depth).*
Access & import/export	*Describe the efforts you have made to access habitats and to collect and import/export your samples in a responsible manner and in compliance with local, national and international laws, noting any permits that were obtained (give the name of the issuing authority,*

the date of issue, and any identifying information).

Disturbance | Describe any disturbance caused by the study and how it was minimized.

Reporting for specific materials, systems and methods

We require information from authors about some types of materials, experimental systems and methods used in many studies. Here, indicate whether each material, system or method listed is relevant to your study. If you are not sure if a list item applies to your research, read the appropriate section before selecting a response.

Materials & experimental systems

n/a	Involved in the study
☐	☒ Antibodies
☒	☐ Eukaryotic cell lines
☒	☐ Palaeontology and archaeology
☐	☒ Animals and other organisms
☐	☒ Clinical data
☒	☐ Dual use research of concern
☒	☐ Plants

Methods

n/a	Involved in the study
☒	☐ ChIP-seq
☒	☐ Flow cytometry
☒	☐ MRI-based neuroimaging

Antibodies

Antibodies used: All sources and amount/dilution for the used antibodies are depicted in full in the manuscript, given the high number of used antibodies

Validation: All sources and amount/dilution for the used antibodies are depicted in full in the manuscript, only comercially available antivodies used

Eukaryotic cell lines

Policy information about cell lines and Sex and Gender in Research

Cell line source(s): No cell lines used

Authentication: Describe the authentication procedures for each cell line used OR declare that none of the cell lines used were authenticated.

Mycoplasma contamination: Confirm that all cell lines tested negative for mycoplasma contamination OR describe the results of the testing for mycoplasma contamination OR declare that the cell lines were not tested for mycoplasma contamination.

Commonly misidentified lines (See ICLAC register): Name any commonly misidentified cell lines used in the study and provide a rationale for their use.

Palaeontology and Archaeology

Specimen provenance: N/A

Specimen deposition: Indicate where the specimens have been deposited to permit free access by other researchers.

Dating methods: If new dates are provided, describe how they were obtained (e.g. collection, storage, sample pretreatment and measurement), where they were obtained (i.e. lab name), the calibration program and the protocol for quality assurance OR state that no new dates are provided.

☐ Tick this box to confirm that the raw and calibrated dates are available in the paper or in Supplementary Information.

Ethics oversight: Identify the organization(s) that approved or provided guidance on the study protocol, OR state that no ethical approval or guidance was required and explain why not.

Note that full information on the approval of the study protocol must also be provided in the manuscript.

Animals and other research organisms

Policy information about studies involving animals; ARRIVE guidelines recommended for reporting animal research, and Sex and Gender in Research

Laboratory animals	all animal experiments followed applicable guidelines and ethics approval. Mice were housed in standard cages in pathogen-free facilities, under a 12-h light/dark cycle. The animals were provided with unrestricted access to food and water. Prior to euthanasia for tissue collection, the animals were fully anesthetized in a CO2 chamber. After spinal reflexes were tested (by pinching the tail, upper and lower limbs with a laboratory tweezer), the animals were subsequently euthanized by cervical dislocation.
Wild animals	not employed, not relevant for this study
Reporting on sex	sex was reported for all animal experiments. For each model, ten transgenic and ten non-transgenic mice were selected and balanced for sex: For each model, a total of 20 transgenic and non-transgenic mice were selected and balanced for condition and sex (TDP-43 model: 5 females vs. 5 males for transgenic and control cohorts; SOD1 model: 5 females vs. 6 males for the control cohort and 5 females vs. 4 males for the transgenic cohort; C9orf72 model: 6 females vs. 4 males for the control cohort and 4 females vs. 6 males for the transgenic cohort; FUS model: 5 females vs. 5 males for transgenic and control cohorts;).
Field-collected samples	not employed, not relevant for this study
Ethics oversight	All animal experiments complied with international and local animal welfare standards and were approved by the respective regulatory organs of the involved research centers: Mario Negri Institutional Animal Care and Use Committee and the Italian Ministry of Health (Prot. No. 9F5F5.143, ministry authorization no. 4/2020-PR;/Prot. No. 9F5F5.250, ministry authorization no. 496/2023; Direzione Generale della Sanità Animale e dei Farmaci Veterinari, Ufficio 6); German Animal Welfare Act (Tierschutzgesetz/ Tierschutz-Versuchstierverordnung, Regierungsbezirke Oberbayern, Prot. Nos. TV 55.2-2532.Vet_03-17-68/TV 55.2-2532.Vet_02-17-106); the (CCD) Centrale Commissie Dierproeven of Utrecht University (CCD license: AVD 1150020171565) and the Dutch animal welfare laws (Wet op de Dierproeven 2014)/European regulations (guideline 2010/63/EU).

Note that full information on the approval of the study protocol must also be provided in the manuscript.

Clinical data

Policy information about clinical studies

All manuscripts should comply with the ICMJEguidelines for publication of clinical research and a completedCONSORT checklist must be included with all submissions.

Clinical trial registration	not relevant for this study
Study protocol	not relevant for this study
Data collection	clinical data from patients selected for the postmortem analysis provided by the brain banks anf followed ethics approval
Outcomes	not relevant for this study

Dual use research of concern

Policy information about dual use research of concern

Hazards

Could the accidental, deliberate or reckless misuse of agents or technologies generated in the work, or the application of information presented in the manuscript, pose a threat to:

No	Yes	
☒	☐	Public health
☒	☐	National security
☒	☐	Crops and/or livestock
☒	☐	Ecosystems
☒	☐	Any other significant area

Experiments of concern

Does the work involve any of these experiments of concern:

No	Yes	
☒	☐	Demonstrate how to render a vaccine ineffective
☒	☐	Confer resistance to therapeutically useful antibiotics or antiviral agents
☒	☐	Enhance the virulence of a pathogen or render a nonpathogen virulent
☒	☐	Increase transmissibility of a pathogen
☒	☐	Alter the host range of a pathogen
☒	☐	Enable evasion of diagnostic/detection modalities
☒	☐	Enable the weaponization of a biological agent or toxin
☒	☐	Any other potentially harmful combination of experiments and agents

Plants

Seed stocks	N/A
Novel plant genotypes	*Describe the methods by which all novel plant genotypes were produced. This includes those generated by transgenic approaches, gene editing, chemical/radiation-based mutagenesis and hybridization. For transgenic lines, describe the transformation method, the number of independent lines analyzed and the generation upon which experiments were performed. For gene-edited lines, describe the editor used, the endogenous sequence targeted for editing, the targeting guide RNA sequence (if applicable) and how the editor was applied.*
Authentication	*Describe any authentication procedures for each seed stock used or novel genotype generated. Describe any experiments used to assess the effect of a mutation and, where applicable, how potential secondary effects (e.g. second site T-DNA insertions, mosiacism, off-target gene editing) were examined.*

ChIP-seq

Data deposition

☐ Confirm that both raw and final processed data have been deposited in a public database such as GEO.

☐ Confirm that you have deposited or provided access to graph files (e.g. BED files) for the called peaks.

Data access links<br>*May remain private before publication.*	N/A
Files in database submission	*Provide a list of all files available in the database submission.*
Genome browser session<br>(e.g. UCSC)	*Provide a link to an anonymized genome browser session for "Initial submission" and "Revised version" documents only, to enable peer review. Write "no longer applicable" for "Final submission" documents.*

Methodology

Replicates	*Describe the experimental replicates, specifying number, type and replicate agreement.*
Sequencing depth	*Describe the sequencing depth for each experiment, providing the total number of reads, uniquely mapped reads, length of reads and whether they were paired- or single-end.*
Antibodies	*Describe the antibodies used for the ChIP-seq experiments; as applicable, provide supplier name, catalog number, clone name, and lot number.*
Peak calling parameters	*Specify the command line program and parameters used for read mapping and peak calling, including the ChIP, control and index files used.*
Data quality	*Describe the methods used to ensure data quality in full detail, including how many peaks are at FDR 5% and above 5-fold enrichment.*
Software	*Describe the software used to collect and analyze the ChIP-seq data. For custom code that has been deposited into a community repository, provide accession details.*

Flow Cytometry

Plots

Confirm that:

- [ ] The axis labels state the marker and fluorochrome used (e.g. CD4-FITC).
- [ ] The axis scales are clearly visible. Include numbers along axes only for bottom left plot of group (a 'group' is an analysis of identical markers).
- [ ] All plots are contour plots with outliers or pseudocolor plots.
- [ ] A numerical value for number of cells or percentage (with statistics) is provided.

Methodology

Sample preparation	N/A
Instrument	*Identify the instrument used for data collection, specifying make and model number.*
Software	*Describe the software used to collect and analyze the flow cytometry data. For custom code that has been deposited into a community repository, provide accession details.*
Cell population abundance	*Describe the abundance of the relevant cell populations within post-sort fractions, providing details on the purity of the samples and how it was determined.*
Gating strategy	*Describe the gating strategy used for all relevant experiments, specifying the preliminary FSC/SSC gates of the starting cell population, indicating where boundaries between "positive" and "negative" staining cell populations are defined.*

- [ ] Tick this box to confirm that a figure exemplifying the gating strategy is provided in the Supplementary Information.

Magnetic resonance imaging

Experimental design

Design type	N/A
Design specifications	*Specify the number of blocks, trials or experimental units per session and/or subject, and specify the length of each trial or block (if trials are blocked) and interval between trials.*
Behavioral performance measures	*State number and/or type of variables recorded (e.g. correct button press, response time) and what statistics were used to establish that the subjects were performing the task as expected (e.g. mean, range, and/or standard deviation across subjects).*

Acquisition

Imaging type(s)	*Specify: functional, structural, diffusion, perfusion.*
Field strength	*Specify in Tesla*
Sequence & imaging parameters	*Specify the pulse sequence type (gradient echo, spin echo, etc.), imaging type (EPI, spiral, etc.), field of view, matrix size, slice thickness, orientation and TE/TR/flip angle.*
Area of acquisition	*State whether a whole brain scan was used OR define the area of acquisition, describing how the region was determined.*

Diffusion MRI ☐ Used ☐ Not used

Preprocessing

Preprocessing software	*Provide detail on software version and revision number and on specific parameters (model/functions, brain extraction, segmentation, smoothing kernel size, etc.).*
Normalization	*If data were normalized/standardized, describe the approach(es): specify linear or non-linear and define image types used for transformation OR indicate that data were not normalized and explain rationale for lack of normalization.*
Normalization template	*Describe the template used for normalization/transformation, specifying subject space or group standardized space (e.g. original Talairach, MNI305, ICBM152) OR indicate that the data were not normalized.*
Noise and artifact removal	*Describe your procedure(s) for artifact and structured noise removal, specifying motion parameters, tissue signals and physiological signals (heart rate, respiration).*

Volume censoring	*Define your software and/or method and criteria for volume censoring, and state the extent of such censoring.*

Statistical modeling & inference

Model type and settings	No MRI data employed.
Effect(s) tested	*Define precise effect in terms of the task or stimulus conditions instead of psychological concepts and indicate whether ANOVA or factorial designs were used.*

Specify type of analysis: ☒ Whole brain ☐ ROI-based ☐ Both

Statistic type for inference (See Eklund et al. 2016)	*Specify voxel-wise or cluster-wise and report all relevant parameters for cluster-wise methods.*
Correction	*Describe the type of correction and how it is obtained for multiple comparisons (e.g. FWE, FDR, permutation or Monte Carlo).*

Models & analysis

n/a	Involved in the study
☐	☐ Functional and/or effective connectivity
☐	☐ Graph analysis
☐	☐ Multivariate modeling or predictive analysis

Functional and/or effective connectivity	*Report the measures of dependence used and the model details (e.g. Pearson correlation, partial correlation, mutual information).*
Graph analysis	*Report the dependent variable and connectivity measure, specifying weighted graph or binarized graph, subject- or group-level, and the global and/or node summaries used (e.g. clustering coefficient, efficiency, etc.).*
Multivariate modeling and predictive analysis	*Specify independent variables, features extraction and dimension reduction, model, training and evaluation metrics.*
