# Supplementary figures and images for: Multiomic ALS signatures highlight subclusters and sex differences suggesting the MAPK pathway as therapeutic target
Source: Nat Commun. 2024 Jun 7;15:4893. doi: 10.1038/s41467-024-49196-y (PMC11161513; doi:10.1038/s41467-024-49196-y)

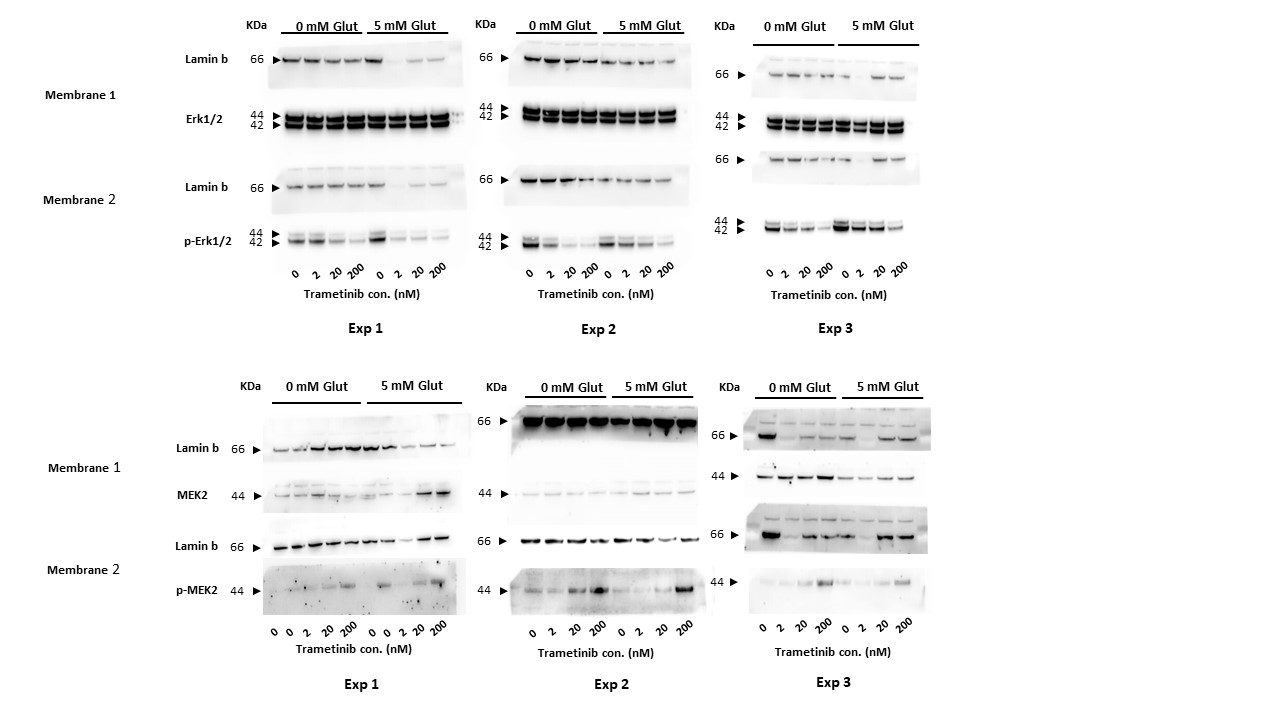

Supplement: Supplementary file 6 — Source Data [file 41467_2024_49196_MOESM6_ESM.zip › Source data files/Uncropped WB membranes phospho ERK MEK.jpg]
